# Supplementary material for: Effectiveness of a Nanohydroxyapatite-Based Hydrogel on Alveolar Bone Regeneration in Post-Extraction Sockets of Dogs with Naturally Occurring Periodontitis
Source: Vet Sci. 2021 Dec 26;9(1):7. doi: 10.3390/vetsci9010007 (PMC8777875; doi:10.3390/vetsci9010007)
Supplement: Supplementary file 1 [file vetsci-09-00007-s001.zip › vetsci-1501427-supplementary.pdf]

Supplemental Table

Supplemental Table S1.

The American Society of Anesthesiologists Physical Status Classification System

ASA Classification	Animal Heath Description
**ASA I**	No systemic illness; healthy animal
**ASA II**	Mild compensated systemic illness (e.g., Hypothyroidism, Obesity)
**ASA III**	Moderate-severe compensate systemic illness (e.g., heart disease, chronic renal failure, liver disease, diabetes mellitus)
**ASA IV**	Severe systemic disease and incapacitated
**ASA V**	Moribund, terminally ill
**Emergency**	Cesarean section, Gastric dilation-volvulus and pneumothorax

Supplemental Table S2.

Anesthesia protocol from Surgery unit, small animal teaching hospital, The Faculty of Veterinary Medicine, Chiang Mai University

Medication	Drug	Dosage
**Fluid therapy**	Acetate ringer's solution	2-10 ml/kg/hr.
**Local anesthesia**	2% Lidocaine hydrochloride (Locana®)	1 mg/kg or 0.3-0.5 ml/site
**Premedication**	Midazolam HCl (Hemalin®)	0.2-0.4 mg/kg IM, IV
	Morphine	0.2-0.5 mg/kg SC
**Induction**	Propofol (Profol®)	4-6 mg/kg Slow IV
**Maintenance**	Isoflurane (Aerrane®)	0.5-2% Inhalation
**Anti-inflammatory drug**	Carprofen (Rimadyl®)	4.4 mg/kg SC
**Antiseptic mouthwash**	Chlorhexidine gluconate	0.12 %

Supplemental Table S3.

The Stage of periodontal disease, Diagnostic indexes and treatment suggestion from the American Veterinary Dental College

Stage of Periodontal disease		Diagnostic index		Treatment suggestion
		**Attachment loss**	**Probing depth (mm)[a]**	
**Normal (PD0)**	Clinically normal	Only without attachment loss	< 3	Routine dental home care
**Stage 1 (PD1)**	Gingivitis	Only without attachment loss	< 3	Routine dental home care
**Stage 2 (PD2)**	Early periodontitis	Less than 25% of attachment loss	< 5	Professional Periodontal therapy (under anesthesia)
**Stage 3 (PD3)**	Moderate periodontitis	Approximate 25-50% of attachment loss	< 7	Professional Periodontal therapy (Under anesthesia)<br><br>The options for treatment includes<br>Dental Extraction<br>Advanced treatment by a veterinary dental specialist
**Stage 4 (PD4)**	Advanced periodontitis	More than 50% of attachment loss	> 7	Professional Periodontal therapy (Under anesthesia)<br><br>The options for treatment includes<br>Dental Extraction<br>Advanced treatment by a veterinary dental specialist

[a] Probing depth is highly variable according to animal size, Attachment loss is a more accurate measurement.
